# Venereal Clinics in U.S.A.

**Published:** 1919-03-01

**Authors:** 


### VENEREAL CLINICS IN U.S.A.

### The Value of State Control

IN England it is gratifying to note the recent appointment of an Inter-Departmental Committee to investigate the dangers of communicable disease incident on demobilization, and to arrange for concerted administration.

The task before such a body is enormous, and, be its actions allowed to progress along truly scientific and economic lines, it has the opportunity of accomplishing one of the finest post-war services. The arrangement is at least a Government recognition of a menace.

Easily, in most diseases, can they deal with the question of precautionary measures, but in connection with venereal infection there are unavoidable complications. The matter has been referred to on these pages in a plea for controlled prophylactic teaching and notification, and it is interesting to read the recent report of Dr. J. D. Robertson on the organisation in Chicago, where, on June 29, 1917, an ordinance was obtained requiring that: (1) The physician must report all cases of gonorrhœa, syphilis, and chancroid; (2) The patient must receive an education circular; (3) If the physician be changed, the fact must be reported to the first; (4) Failure to attend for treatment must be reported to the Health Department which at once investigates. A municipal clinic and hospital were found essential for the enforcement, and these, in the first ten months, dealt with 5,258 cases. Outside measures were many and varied. Quack advertisements to the number of 1,500 were removed in the town and 3,500 educational posters substituted; known prostitutes were arrested by the police and examined in the first place at a general hospital, while druggists were instructed against treatment and counter-prescribing. To use Dr. Robertson's own words, "education and the big stick" were the necessities, and with them, within twelve months, he found the disease well under control.

Next, referring to another American communication, under the State Law of Massachusetts of 1918 all cases must be reported to the State Department of Health first by number and, if failing to keep up treatment satisfactorily, by name also. The State makes itself responsible for their supervision and, in Boston, co-operation between the Health Department and the clinic is complete. Under a central control are placed separate departments with distinct but co-ordinated duties, namely: (a) The clinical work of diagnosis and treatment carried to completion; (b) Public health work to deal with prevention of spread through personal, family, or occupational contacts or through vice, and also to protect the community against those who, if insufficiently treated, are likely to become public charges, due to physical breakdown; (c) Social work to prevent the break-up of families, due to existence of disease, and to aid when necessary in maintaining employment. Each has the support of the official mandate and a high degree of efficiency is claimed for their work.

Can we not profit by these experiences of our most progressive Ally with her more drastic legislation?

